# Hidradenitis Suppurativa and Recurrent Peripheral Ulcerative Keratitis: A Case Report

**DOI:** 10.7759/cureus.77494

**Published:** 2025-01-15

**Authors:** Klara Ambaye, Victor Cox, Abhishek Naidu, Aruoriwo Oboh-Weilke

**Affiliations:** 1 Ophthalmology, Georgetown University School of Medicine, Washington, DC, USA; 2 Ophthalmology, MedStar Georgetown University Hospital, Washington, DC, USA

**Keywords:** cornea, hidradenitis suppurativa, inflammatory eye disease, peripheral ulcerative keratitis, recurrent keratitis

## Abstract

This case report highlights a rare presentation of a hidradenitis suppurativa (HS) flare occurring concurrently with peripheral ulcerative keratitis (PUK) in a patient with a longstanding history of HS. HS is a chronic inflammatory skin condition known to be associated with various ocular manifestations, including inflammatory eye diseases (IEDs) such as anterior uveitis. Although the link between HS and IED is well documented, the association between HS and PUK is less frequently reported. Previous cases have demonstrated improvement in keratitis among HS patients using treatments ranging from topical steroids to systemic immunosuppressive therapies. In this case, the patient’s symptoms resolved with a combination of topical and oral steroids, supporting the hypothesis that HS may underlie the development of PUK in such cases. This report adds to the growing body of evidence connecting HS with ocular complications and underscores PUK as a rare but significant manifestation of the disease.

## Introduction

Hidradenitis suppurativa (HS) is a chronic inflammatory skin condition characterized by recurrent painful nodules, abscesses, and sinus tracts, primarily affecting areas rich in apocrine sweat glands [1]. Although rare, the association of HS with ocular conditions and its potential role in the development of inflammatory eye diseases (IEDs), such as anterior uveitis, has been reported in several studies [2]. In a study of 236 patients with HS, Lee et al. found that 10% had documented IED, despite most not having any other inflammatory or autoimmune conditions [2]. However, specific cases of HS associated with peripheral ulcerative keratitis (PUK) have been described only sporadically in the literature [3-6]. Previously reported cases showed improvement in keratitis among HS patients using various immunosuppressive therapies, including topical prednisolone, oral cyclophosphamide, and biologics like adalimumab [3-6]. Here, we present a case of a patient with longstanding HS and recurrent episodes of PUK, aiming to contribute further evidence to the potential association between HS and IED.

## Case presentation

A 54-year-old African American male with a medical history of hypertension, diabetes, a 15-year history of HS, and recurrent corneal ulcers presented to the emergency department with a two-day history of worsening eye pain and blurry vision. Two years prior, during an episode of a corneal ulcer in the left eye, he had been started on tobramycin/dexamethasone drops but was subsequently lost to follow-up. The following year, he was admitted for a corneal ulcer in the right eye with concern for perforation. At that time, he was noted to have active inflammation of the buttocks from HS and underwent an extensive infectious and autoimmune disease workup for his corneal disease. Testing for hepatitis A, B, and C, HIV, *Treponema pallidum*, tuberculosis, antinuclear antibodies (ANA), antineutrophil cytoplasmic antibodies (ANCA), anti-double-stranded DNA, proteinase 3, and myeloperoxidase was all negative.

**Figure 1:**
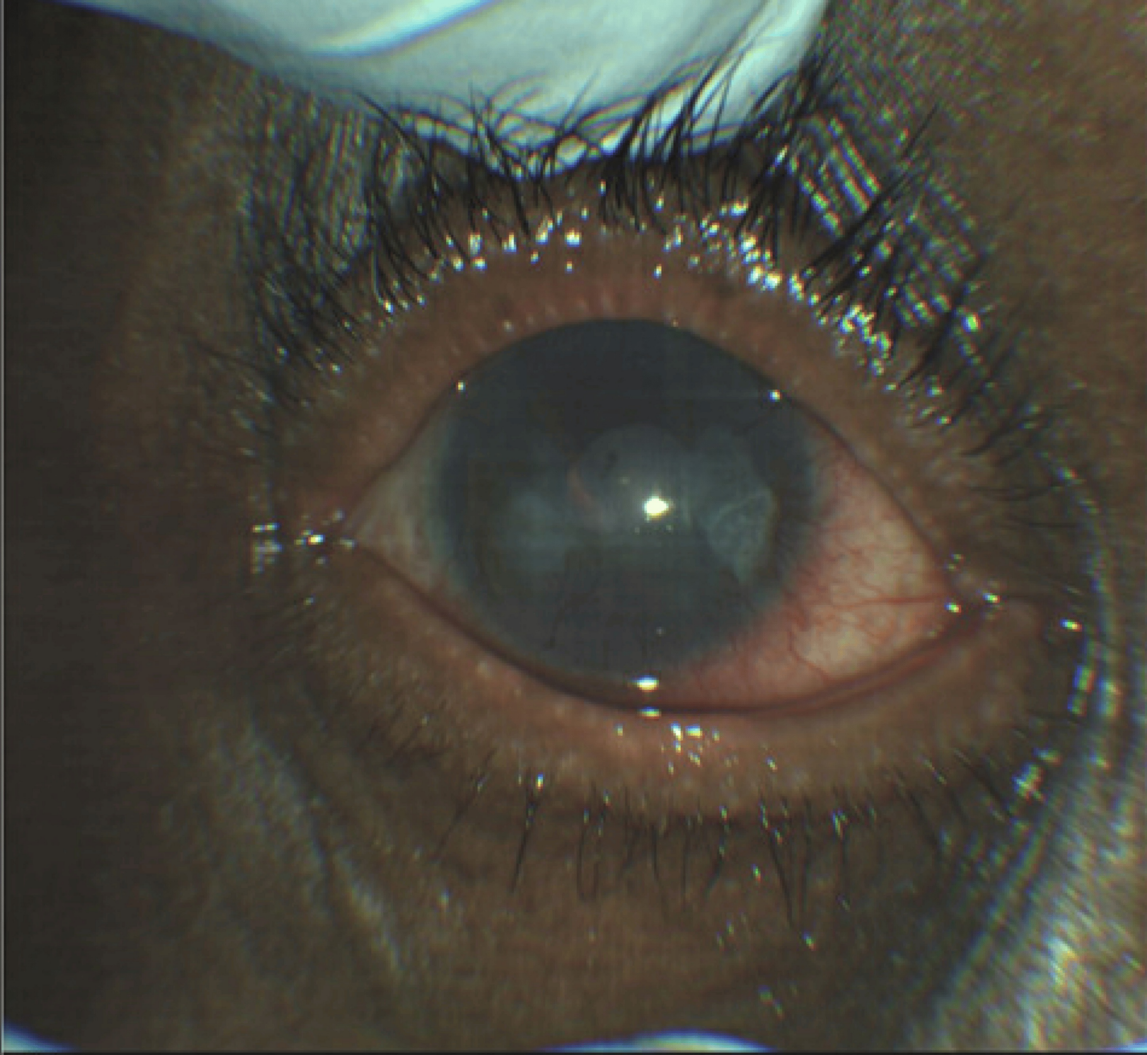
External photograph of the right eye at presentation, captured using a ZEISS CLARUS 500 Fundus Camera (Oberkochen, Germany)

**Figure 2:**
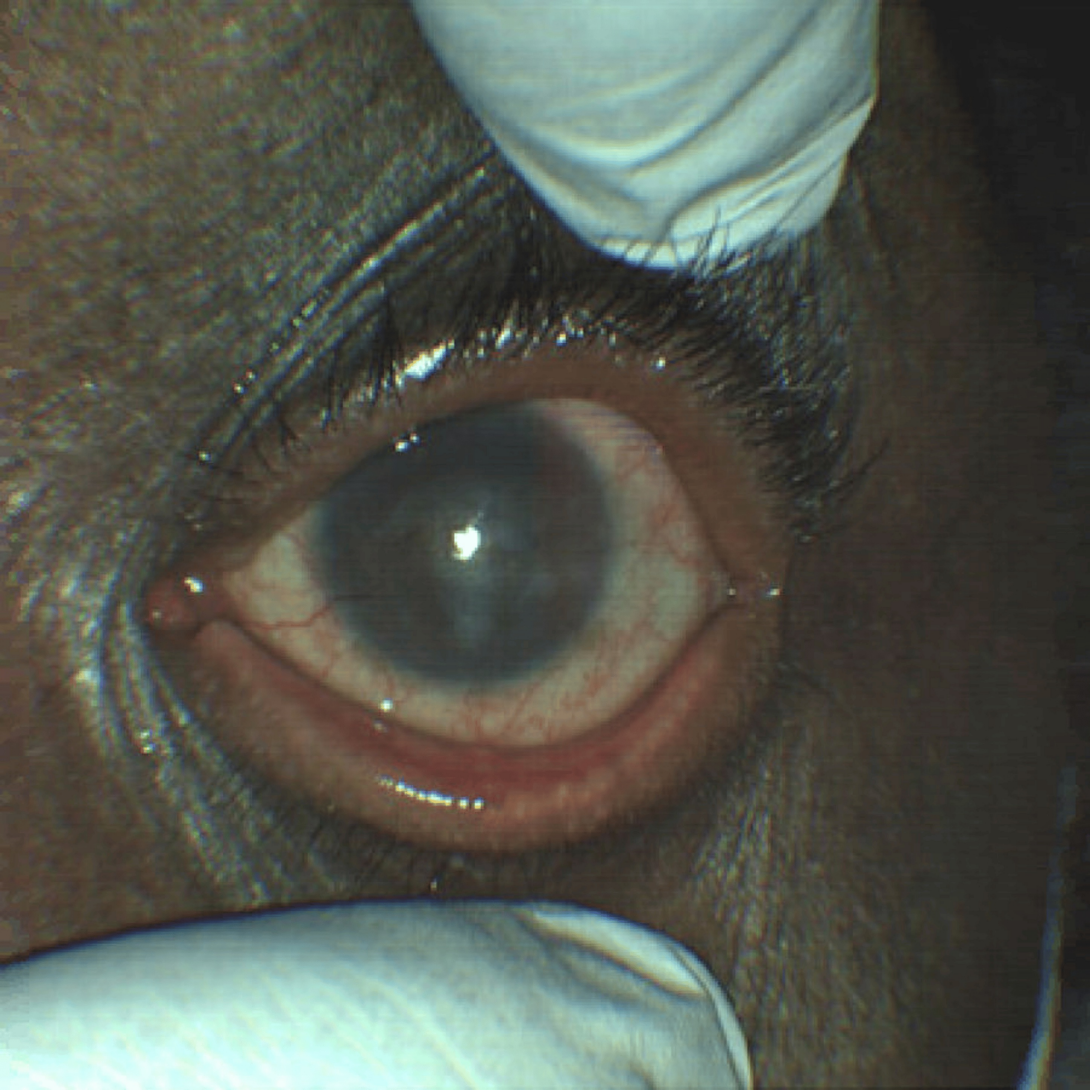
External photograph of the left eye at presentation, captured using a ZEISS CLARUS 500 Fundus Camera (Oberkochen, Germany)

During his emergency department presentation, his initial vitals were as follows: temperature 37.4°C, heart rate 66 bpm, blood pressure 135/85 mmHg, and oxygen saturation 100% on room air. Ophthalmic examination revealed a visual acuity of 20/200 in the right eye and hand motion in the left eye, with intraocular pressures of 14 and 16 mmHg, respectively. Pupils were equal, round, and reactive bilaterally; extraocular movements and visual fields were full; and no relative afferent pupillary defect was noted. Examination showed meibomian gland dysfunction with scurf on the eyelids of both eyes, 2-3+ conjunctival injections, epiphora, and non-blanching scleral vessels. The right cornea demonstrated diffuse corneal neovascularization, a 1.5 × 1.5 mm ulcer with 60% thinning inferonasally, prominent perilimbal vessels, and conjunctivalization. The left cornea showed similar findings, with diffuse corneal neovascularization, prominent perilimbal vessels, and conjunctivalization. Both anterior chambers were deep and quiet. These findings were consistent with noninfectious keratitis, such as PUK (Figure 1, Figure 2).

On skin examination, the patient had active pustular lesions in the groin and buttocks, consistent with active HS. He was started on topical erythromycin 0.5% ophthalmic ointment four times daily, moxifloxacin 0.5% ophthalmic solution four times daily, prednisolone acetate 1% ophthalmic suspension twice daily, oral valacyclovir 500 mg three times daily, vitamin C 100 mg daily for his corneal findings, and oral doxycycline 100 mg daily for HS. Another infectious workup, including testing for HIV, syphilis, HSV, tuberculosis, Lyme disease, gonorrhea, and chlamydia, was negative. Prednisolone and moxifloxacin drops were discontinued, and tobramycin/dexamethasone 0.3-0.1% drops four times daily and oral prednisone 40 mg daily were started. An inflammatory disease workup, including ANA, ANCA, rheumatoid factor, and anti-cyclic citrullinated protein (CCP) testing, was negative, and a chest X-ray showed no hilar lymphadenopathy. However, the erythrocyte sedimentation rate and CRP levels were elevated.

Given the bilateral involvement, symptomatic improvement with oral and topical steroids and doxycycline, and the exclusion of infectious and autoimmune causes, poorly controlled HS was considered the underlying etiology of the patient’s PUK. Seven days after admission, the patient reported significant improvement in pain, photophobia, and visual acuity. Examination revealed improvement in visual acuity to 20/70 in the right eye and 20/400 in the left, with regression of stromal haze and diffuse corneal neovascularization in both eyes. By day 10, the patient denied ocular pain, and his visual acuity had further improved to 20/40 in the right eye.

The patient was discharged 11 days later on an oral prednisone taper, topical tobramycin-dexamethasone 0.3-0.1% drops four times daily, oral doxycycline 100 mg daily, and vitamin C 2 g daily. Unfortunately, he was lost to follow-up thereafter.

## Discussion

PUK is characterized by inflammation that causes epithelial defects and destruction of the corneal stroma, leading to peripheral corneal ulceration [7]. Although reports of concurrent HS flares and PUK are exceedingly rare, a few cases with similar presentations have been documented [3-6]. This report adds another intriguing example of a patient experiencing recurrent keratitis during active HS. As in previously reported cases, the absence of significant findings in inflammatory and infectious disease workups, combined with improvement following immunosuppressive therapy, suggests that HS may be the underlying cause of the observed PUK [3-6].

The differential diagnosis for PUK includes ANCA-associated vasculitides such as granulomatosis with polyangiitis, microscopic polyangiitis, and eosinophilic granulomatosis with polyangiitis, as well as rheumatologic diseases like rheumatoid arthritis and systemic lupus erythematosus and inflammatory bowel disease [7]. A comprehensive workup for PUK should include ANA, anti-CCP, and ANCA testing to rule out these conditions, alongside investigations to exclude infectious causes [7]. In our patient, results for ANCA, anti-CCP, and infectious workups were unremarkable, and no clinical signs or symptoms indicated the presence of these pathologies, thereby reducing the likelihood of vasculitides, rheumatologic diseases, or infections as drivers of the ocular findings.

The precise connection between HS and PUK remains unclear but is hypothesized to arise from shared mechanisms of immune dysregulation [8]. While not extensively studied, there are reports of patients with HS presenting with ocular manifestations, such as anterior uveitis, in the absence of comorbid inflammatory conditions [2]. This pattern suggests that HS may serve as a plausible etiology for IEDs, including cases presenting as PUK [8]. Our case highlights the need to recognize potential ocular complications of HS, with PUK as a possible manifestation. However, further clinical evidence is needed to establish a definitive association.

## Conclusions

PUK is an inflammatory condition characterized by corneal ulceration and epithelial defects, often associated with underlying immune dysregulation. While cases of PUK occurring concurrently with active HS are rare, they have been documented. In this case, the patient’s recurrent keratitis improved with immunosuppressive therapy, supporting the hypothesis that HS may be a contributing factor to PUK. Although the exact relationship between HS and PUK remains unclear, the occurrence of ocular manifestations in patients with HS underscores the need for further investigation into this potential association.
